# Making Informed Choices about Microarray Data Analysis

**DOI:** 10.1371/journal.pcbi.1000786

**Published:** 2010-05-27

**Authors:** Mark Reimers

**Affiliations:** Department of Biostatistics, Virginia Commonwealth University, Richmond, Virginia, United States of America; Whitehead Institute, United States of America

This *PLoS Computational Biology* tutorial was presented at ISMB 2008

This article describes the typical stages in the analysis of microarray data for non-specialist researchers in systems biology and medicine. Particular attention is paid to significant data analysis issues that are commonly encountered among practitioners, some of which need wider airing. The issues addressed include experimental design, quality assessment, normalization, and summarization of multiple-probe data. This article is based on the ISMB 2008 tutorial on microarray data analysis. An expanded version of the material in this article and the slides from the tutorial can be found at http://www.people.vcu.edu/~mreimers/OGMDA/index.html.

## Introduction: Why Is Data Analysis Still an Issue?

High-throughput methods are revolutionizing biological research, and numerous published articles describe innovative insights obtained through analysis of microarray data. Microarray technologies are now available for measuring gene expression, DNA copy number, methylation, chromatin state, protein binding, SNPs, and other aspects of gene physiology. To address the needs of many biomedical researchers who do not often have the resources to perform sophisticated data manipulation, several companies and institutions have prepared pre-packaged software to guide the researcher through and perform all the steps of standard microarray analysis. Commercial packages include Genespring, Nexus (from Biodiscovery), GeneSifter (from Geospiza), Expressionist (from Genedata), Partek Genomics Suite, and many others. Several institutional packages are described at the end of this article. With such packaged software readily available, who really needs to think about microarray analysis or to collaborate with array analysis specialists?

The premise of this review is that innovative microarray analyses are rarely straightforward and that most analyses present problems and opportunities that are not readily identified or adequately addressed by packaged, comprehensive software. For example, one common problem in my experience is that common quality assessment (QA) practices may be unable to eliminate all seriously compromised chips. Furthermore, the current best practices for expression arrays don't work well for many of the new kinds of arrays (see Text S1, section S2). Certainly, commercial software has valuable uses; e.g., the quality and flexibility of commercial graphics programming for data visualization that outstrips that of open-source software. However, this review proposes a Malthusian maxim for microarrays: that the number of potential complications in high-throughput biology grows exponentially, while the expertise embodied in packaged software has grown only linearly. Thus, a researcher must know enough about high-throughput methods to ascertain when commercialized software can be helpful and when an expert in microarray analysis should be consulted.

**Figure 1:**
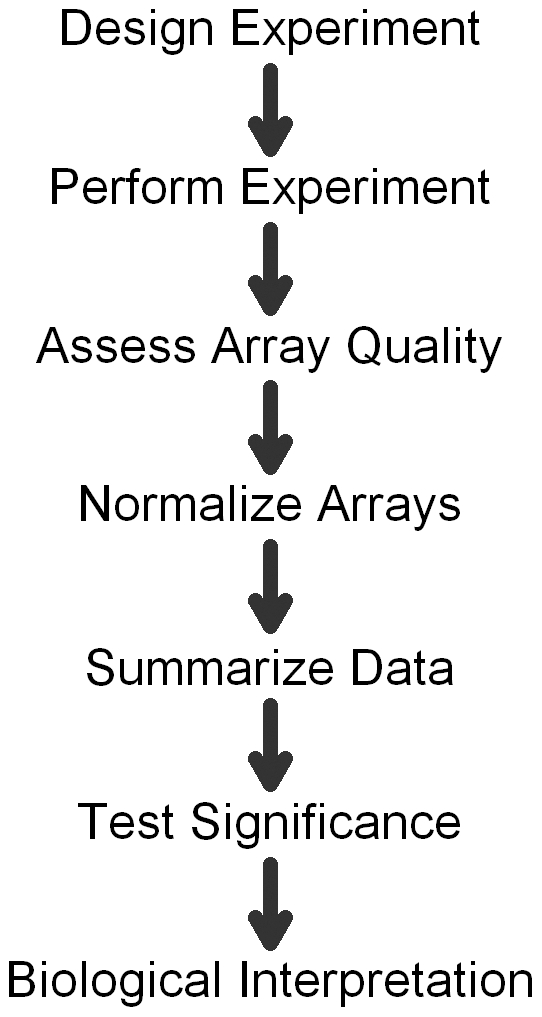
Steps in a typical microarray analysis.

Most data analyses consist of the steps outlined in Figure 1. This review will follow these steps. The last two steps (significance tests and biological interpretation) and exploratory analysis are not covered here. A complementary approach to some of the early stages of a study was presented recently in [1].

## Experimental Design

Researchers know good data depends on good experimental design. There are several design issues that benefit from statistical thinking, but the most prominent issue is whether there will be enough samples to find most of the genes that are changed. Most researchers are aware of the multiple comparisons issue; when they perform a straightforward power calculation, they know to set the target significance threshold much lower than it would be for single tests. However, the actual level of significance one would need to detect a significant difference after standard Bonferroni multiple comparisons adjustments to the *p*-value would be so small that an experiment would require far too many samples to be practical. For this reason, in practice most researchers use the false discovery rate (FDR) [2] to assess significance in data analysis. It makes sense, therefore, to perform power calculations using FDR, but such a calculation is not so easy to perform. Another approach to power estimation is to make an analogy with published data to estimate the power of one's own study; such an approach is implemented in the Microarray Power Atlas ([3] and http://www.poweratlas.org).

A general principle of experimental design is to make comparisons under circumstances as closely matched as possible. A second issue that has tripped up many researchers is that arrays processed at different times or by different technicians often show pronounced batch differences [4], [5]. For example, there may be different dynamic ranges in different batches, or certain subsets of probes may show greater signal independent of biological differences. Thus, it is important to try to process all the arrays under as close to identical conditions as possible, or, if that is impossible, to randomize the assignment of samples to processing times. Variations in ozone concentration make a significant difference (R. Lucito, E. Ljungstrom, personal communications; [6], [7]).

In designing two-color array experiments, the most common advice is to make contrasts that are the most informative [1], [8]. Such a design helps to maximize the information available in a fixed number of arrays. For various reasons, such as flexibility in the face of hybridization failures, and in order to allow comparisons between present and possible future experiments, many experimenters choose to co-hybridize both case/treatment and control samples with a common reference sample. In keeping with the general principle in the previous paragraph, it is better not to co-hybridize samples that are expected to differ markedly, because extreme ratios of gene expression, far from one, usually have much larger errors. Therefore, if one is using a common reference, it is better to use a reference from a tissue similar to the samples under study, rather than a reference from a different tissue.

## Quality Assessment

Once an experiment has been designed and performed, the next question is how to decide which data are worth the effort to analyze. Data are only as good as the samples, and many researchers scrupulously check RNA quality before hybridization. However, few researchers are able to check the subsequent labelling and the physical chemistry that occurs during hybridization. In typical lab practice focused on characterization of a single gene, researchers draw on their experience to optimize conditions for a particular RNA target. However, it isn't possible to optimize conditions for all probes simultaneously on an array. Microarray measures represent a dynamic balance among many competing processes, and many factors can shift these processes noticeably; for example, the ratio of off-target hybridization to true signal from each probe depends on the relation among the hybridization temperature, ionic strength, and the thermodynamic characteristics of the probe. Sometimes technical faults or differences in technique peculiar to one array can give very odd results without any obvious indication in the QA metrics that are routinely monitored in many labs. In my experience with several labs at leading institutions, arrays have slipped by with particles of dust or scratches on a chip, air bubbles in the hybridization, or wipe marks (or even fingerprints!) on a glass cover slip. These faults are often not visible to the naked eye but can make a big difference to data quality.

**Figure 2:**
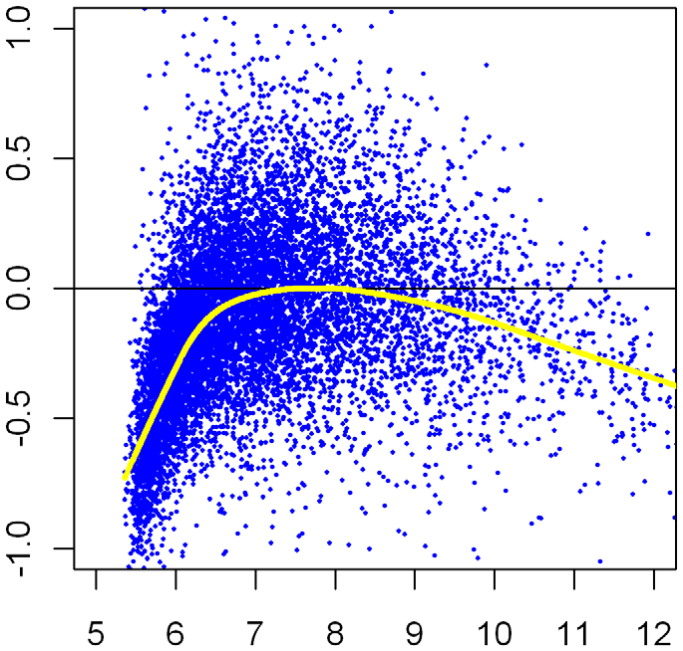
Plot of log ratio of intensity of an Affymetrix array. This plot represents deviations of measures from chip GSM25526 (from GSE2552 in GEO) relative to the average across all chips (on the vertical axis) plotted against that average (in log2 units on the horizontal axis) as a technical variable. The black line indicates no trend; a loess fit to the trend is plotted in yellow. Clearly, the deviation of the trend from 0 is bigger than the standard deviation of the variation around the trend, a sign of a significant technical artifact.

One general statistical approach to address QA issues is to compare each chip to an ideal reference and look for unusually large departures from the ideal that seem correlated with known technical variables [9]–[11]. In practice we don't know what the ideal reference values should be, but for data sets drawn from similar tissue types a robust mean of each probe value across all arrays approximates the ideal for that probe reasonably well. A good way to look for technical faults is to plot deviations of values on one chip from their averages across many chips against any technical variable [11]. For samples all taken from one tissue type, most intensities are roughly constant, and so average probe intensity is a sensitive indicator of probe saturation and quenching. Figure 2 shows a plot of log deviation against average probe intensity. The average bias is indicated as a function of intensity. Note that the bias is very strongly negative for the lower intensities; in fact, bias accounts for much more difference from other chips than all other sources of variation, which includes biological differences. A well-prepared array from an unusual sample may well show substantial dispersion around the bias curve, but the variation in the bias curve will be smaller than the standard deviation of the dispersion.

**Figure 3:**
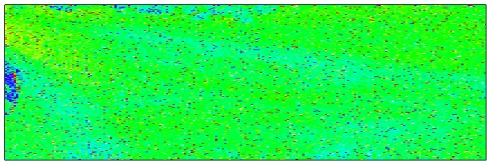
False-color images of an Agilent array (AG1-Lab2-C1 from the MicroArray Quality Control project). Green pixels represent probes whose value on this array is close to their average values across all samples. Red pixels represent probes whose values are more than 1.41 (square root of 2) times their average values across all samples, while dark blue pixels represent probes whose values are less than 0.71 (reciprocal of square root of 2) times those averages.

**Figure 4:**
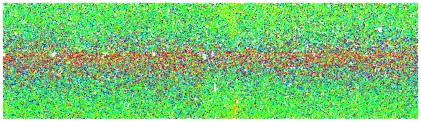
False-color image of an Illumina array. Colors represent how the signal from a particular bead deviates from the average signal from that bead type across the array. The color scale is as for Figure 3.

Some of the most striking images come from representing variability across the physical extent of a chip. Figures 3 and 4 show representations of variability across the chip surface on an Agilent spotted array and on an Illumina array. Figure S1 in Text S1 shows variation across spotted arrays and on Affymetrix chips [9]. No technology is immune from these kinds of artifacts, although the measures from multi-probe technologies, such as Affymetrix and Illumina, and to some extent NimbleGen, are more robust to artifacts, because many probes for a particular gene will lie outside the area affected by a regional artifact. A more sophisticated approach to spatial variability, designed specifically for Affymetrix arrays, uses the idea of fitting a linear model to the profile of each probe set separately. This approach eliminates from the QA metrics differences between chips due to true differences in gene expression, such as those that occur in Figure 3. A “rogues' gallery” of images from several public Affymetrix data sets is at http://www.plmimagegallery.bmbolstad.com/.

Numerous public domain tools exist for quality assessment of array data. Bioconductor (http://www.bioconductor.org) features several packages for Affymetrix, such as affyQCReport, and for spotted arrays, such as arrayQuality and arrayQualityMetrics. Some code examples used in this paper will be posted at http://www.people.vcu.edu/~mreimers/OGMDA/index.html.

## Normalization

Many researchers have tested reproducibility of array data by assaying the same samples on the same platform at different times or by employing different technicians. The results are often unsettling; when sample profiles from such studies are compared, the technical differences are often comparable to the biological differences [12]. Identification of key factors in reproducibility is challenging because data analysts generally don't have access to records of the procedures with sufficient detail to identify crucial differences in technique. And if such differences were in fact known in advance, technicians would attempt to minimize them. The aim of normalization is to compensate the measures for the effects of the differences in procedures among the samples being compared without delving into exactly what the crucial technical differences actually were.

A key decision researchers must make, with consequences for normalization, is on what scale to analyze their data. It is common practice to transform to a logarithmic (usually base 2) scale. The principal motivation for this transformation is to make variation roughly comparable among measures that span several orders of magnitude. This often works as intended; however, such a transformation may actually increase variation of the low intensity probes relative to the rest. In particular, when a measure can be reported as zero, the logarithm isn't defined. A simple remedy is to add a small constant to the measures before taking the logarithm. A more sophisticated approach is to use a non-linear variance-stabilizing transform; one such simple transform is *f(x) = *ln*( (x+ (x^2^+c^2^)^1/2^/2)*, where *c* is the ratio of the constant portion of the variance to the rate of increase of variance with intensity. For more details and options, see [13]–[15].

A second major decision is whether to use a background compensation, and if so, which method to use. Many assays in molecular biology, including early radioactivity-based array assays for gene expression, show an ubiquitous background signal, onto which is added specific signal from the gene of interest. The situation with microarrays is more complex. Some kinds of scanners and dyes clearly show luminescence across the whole array that seems to be added onto the signals. This kind of background can be estimated from non-probe areas of a chip nearby each probe. In many other types of array, the probes seem to be completely opaque and do not return luminescent signal from the same causes as the surrounding areas. In most arrays, cross-hybridization to probes (which depends on the probe sequence) is a bigger source of background than any uniform physical cause, such as may be inferred from the areas surrounding each probe. In my experience, it is rare that a simple background correction brings a substantial improvement in accuracy (as measured, say, by similarity of replicate chips). Similar results were found by [16]. However, sometimes background compensation may be advantageous, and in those cases some methods are better than others (see [16], [17] for more details).

Once decisions about scale and background are made, researchers often compare the overall distribution of measures on their chips. For microarray pioneers in the late 1990s, the most obvious differences between chips were that some arrays had much brighter scans than others. These differences in measures seemed most likely due to technical differences during the procedures rather than wholesale changes in gene expression; such differences could be explained by variations in the amount of cDNA that was hybridized, by differences in the efficiency of the labelling reaction, and/or by different scanner settings. The simplest compensation for such technical differences was agnostic about the cause of the difference: divide all the values on each chip by the mean over that chip. This normalization made the mean value of all gene measures on each chip to be the same; for two-color arrays, this normalization made the average log ratio between channels on the same chip to be zero. Several variations on this procedure are current: for example, Agilent recommends that the 75th percentiles of intensity distributions be aligned across arrays [18]. This makes some sense if one thinks that in a typical tissue about half the genes are actually expressed; hence, the 75th percentile would be the median of the expressed genes. In our experience, aligning the 75th percentiles doesn't actually perform much better than aligning the medians, and in fact loess or quantile normalization (see below) are much better [19], [20].

The next development in array normalization came in 2001 when Terry Speed and co-workers noticed residual bias in two-color log ratios depending on average intensity; this bias could be seen by plotting the log ratio (log(R)/log(G)) against the average brightness in the two channels. The same bias can be seen in one-color arrays by plotting the intensities on one chip against the intensities averaged across chips as a reference as shown in Figure 2. Terry Speed and co-workers [21] proposed estimating the bias by a non-parametric curve, known as a local regression (loess). The values of log ratios are adjusted by subtracting the estimated bias (the height of the loess curve) at the same average brightness. Such treatment improves most chips but cannot fully compensate for an extreme intensity-dependent bias such as that shown in Figure 2. The method introduced in [21] is now known as “loess normalization”. Loess normalization operates on chips individually, but was intended to make measures comparable across chips as well. Further investigation identified some biases between chips. Hence, there is now a distinction between “within-chip” and “between-chip” (or “across-chip”) normalization. Often, within-chip normalization may be a first step before, or a part of, between-chip normalization.

By 2003, statisticians were developing more complex normalizations. Some statisticians noticed that there were pronounced differences in the loess curves fit to log ratios in different regions of the same chip; they tried to fit separate loess curves to each set of probes produced by a common print tip of a robotically printed cDNA array. Others tried to fit two-dimensional loess surfaces over chips. Further complications included estimating a clone order effect, and re-scaling variation within each print-tip group [22], [23]. In 2003, Benjamin Bolstad, one of Terry Speed's students, proposed cutting through all the complexity by a simple non-parametric normalization procedure, at least for one-color arrays [24]. He proposed shoe-horning the intensities of all probes on each chip into one standard distribution shape, which is determined by pooling all the individual chip distributions. The algorithm mapped every value on any one chip to the corresponding quantile of the standard distribution; hence the method is called “quantile normalization.” This simple between-chip procedure worked as well as most of the more complex procedures that were current at the time, and certainly better than the regression method, which was then the manufacturer's default for Affymetrix chips. This method was also made available as the default in the *affy* package of Bioconductor, which has become the most widely used suite of freeware tools for microarrays (see http://www.bioconductor.org). For all these reasons, quantile normalization has become the normalization procedure which I see most often in papers.

While quantile normalization is a simple, fast, one-size-fits-all solution, it engenders some problems of its own. For example, the genes in the upper range of intensity are forced into the same distribution shape; such shoe-horning reduces biological differences as well as technical differences. A recent adjustment to the quantile procedure in the latest versions of the *affy* package fixes that problem. A second issue is more subtle. For reasons that are still not entirely clear, the errors in different sets of probes are highly correlated [12], [25]. For probes for genes that are in fact not expressed in the samples under study, these correlated errors comprise most of the variation among chips. When quantile normalization acts on these probes, the procedure preserves this apparent but entirely spurious correlation among low-intensity probes and sometimes seems to amplify that correlation. Hence, sophisticated data mining methods that depend on subtle analysis of correlations may pick up spurious relationships [26]. Finally, quantile normalization explicitly depends on the idea that the distribution of gene expression measures does not change across the samples. This assumption is unlikely to be true when testing treatments with severe effects on the transcription apparatus or studying cancer samples with severe genomic aberrations.

Despite these problems, quantile normalization seems to offer the best mix of simplicity and effectiveness of all the general methods for normalization that have appeared in the past six years [19]. It is widely used for multiple-probe oligonucleotide arrays, such as Affymetrix arrays, where it is applied at the probe level. Some people apply quantile normalization at the summary level for Illumina arrays [27]. In my experience, a quantile normalization of both channels in a two-channel microarray is at least as good as, and sometimes better than, the standard loess normalization for these arrays. Perhaps the next stage in normalization will need to address the technical causes of variation. Since each kind of technical variation affects many probes, such an approach may also address the problem of spurious correlations.

Recently, several papers have appeared that address the issue of identifying and compensating batch effects. The comBAT method [5] uses an empirical Bayes methodology: that is, it assumes that the batch effects induce fairly similar deviations in the majority of genes. In my opinion, such an assumption is too strong. However, the author provides an easy software package in R to implement the method. The method of [28] allows for substantially different effects in different genes, and infers a batch structure, which may reflect differences in processing unknown to the data analyst. The paper [28] provides some compelling examples in the field of the genetics of gene expression, showing that systematic technical effects lead to the (false) impression of systemic biological effects, unless some correction is performed, and suggests a method for addressing such effects. The method of [29] infers covariates, which may be affecting many genes simultaneously, using an algorithm related to principal components analysis (PCA).

All of these methods indicate a renewed interest in addressing systematic variation. While these papers do not describe their methods for compensating systematic effects as performing “normalization,” that is in fact what normalization is supposed to do: compensate systematic technical variation. In my opinion, several methods under development will make this approach even more effective and accessible. Naturally, I think two good ones will be [30], [31].

## Summarization

The original idea behind the multiple probe oligonucleotide arrays manufactured by Affymetrix and NimbleGen is that many probes targeting a single gene (a “probe set”) yield many measures; in principle, the average of those measures should give a better estimate of gene expression than any single measure. The reality is more complex, and statisticians have enjoyed considerable debate over how best to construct single expression estimates based on multiple-probe hybridization data. All of these debates presume that the probes in a probe set match a common unique transcript. In light of current knowledge about splice variation and alternate termination, that assumption seems unlikely to be true, although both Affymetrix and NimbleGen did make a reasonable effort to design probe sets to match the specific splice variants that were known at the time of their chips' design. Hence, the signals from one probe set may not all measure the same population of transcripts. One way a user can assess whether this is the case is to plot measures from all probes of one probe set across a set of samples. In my experience, about half the Affymetrix probe sets show consistent changes of all probes in a probe set across samples. In recent years, several authors have attempted to remap Affymetrix probes to ensure that all probes map to the same transcript [32], [33].

The “multi-chip” methods, such as RMA (Robust Multichip Average), are summarization schemes inspired by studying the covariation of probes in a probe set across a set of samples [34]–[36]. The motivation for multi-chip methods comes from reasoning that the signal from one probe in a probe set should depend both on the amount of that transcript in the sample and on the specific affinity of the probe for that transcript. Therefore, although probe signals may differ on any one chip, the signal from each probe should change by the same factor between chips where the amount of transcript in the samples differs.

Comparisons of processing algorithms for oligonucleotide data have shown that the multi-chip methods, which employ comparisons of probe signals across chips, generally have the best signal-to-noise ratio. There is still considerable debate over exactly which multi-chip methods are optimal. Rafael Irizarry has organized a Web-based comparison tool, the AffyComp project (http://affycomp.biostat.jhsph.edu/ and [37]), based on high-quality spike-in data sets published by Affymetrix. In this comparison the gcRMA method, which estimates the non-specific hybridization background of each probe based on sequence, comes out on top. In my opinion, the very well-performed hybridizations done in the manufacturer's own facility are not typical of results in most labs. In particular, I see evidence that the pattern of non-specific hybridization varies substantially between chips in the same experiment, for example by comparing the ratios of PM and MM across chips. Furthermore, when analyzing Affymetrix data produced by typical core facilities, I often find more variability between replicates when processing the raw data by gcRMA than by RMA. Therefore, I prefer to use the more robust plain vanilla RMA.

## Other Array Types

Many of the same issues (QA, normalization, and summarization) arise for new types of arrays; however, many of the methods that have worked well for expression arrays don't apply well to the new array types. Some details of normalization for several new array types are included in the Text S1 section S2.

## Next Steps

Most researchers want the chance to explore their data, to discover unexpected patterns beyond the ideas that informed the study design. Two commonly used methods are clustering and PCA. Clustering is useful for discovering groups of genes with similar expression patterns across a wide range of biological conditions. Alternatively, clustering can be a first step toward identifying molecular sub-types of a complex diagnosis such as cancer [38]. Another exploratory tool is PCA and its relative, correspondence analysis [39], [40]. These methods aim to construct linear combinations of the variables (“components”) that can summarize much of the information in all gene measures across the samples. Space precludes an adequate discussion of these approaches; for more information on clustering, consult chapter 12 of [39] and [41]–[43]. A nice package for multivariate analysis specifically addressing microarray data is MADE4 [44].

Often the goal of a study is either to identify differentially expressed genes or to make effective clinical predictions. Space constraints prevent an adequate exposition here of significance testing and the reader is referred to [21], [45]–[51].

There are a variety of important issues to address in classification. Probably the best single general reference on this topic is [52].

## Prospects for the Next Five Years

Both genomic technology and methods for analysis are in rapid flux. Some issues, such as normalization and summarization, which are important for microarrays, may be addressed by very different approaches with the new technologies. Other issues and approaches seem likely to be more permanent, such as significance testing and exploratory analysis.

Researchers are frequently interested in biology that involves many types of molecules, but DNA/RNA measures are the most accessible with current technology. So, when analyzing genomic data, many researchers are looking at the shadows of the biological processes of interest, e.g., protein activation, which may be better reflected by another technology, e.g., protein arrays, when they become available. However, many of the same analysis issues will arise when protein arrays finally become operational. The technology will be sufficiently different that we may need some new methods for normalization. However, it seems likely that many of the methods worked out for assessing significance of changes in genomic analysis will apply directly to protein or other high-throughput assays.

A current challenge for both basic research and clinical investigation is the integration of multiple data types: expression, genotype, and epigenetic data. All of these may have relevance to predicting clinical outcomes. A number of researchers are proposing methods for combining these types of information [53].

Many researchers are enthusiastic about the prospects for using high-throughput sequencing (HTS) in genomic studies. Some researchers expect that HTS technology will banish the shadows of technical variability that have clouded microarray studies. However, several studies suggest that there is considerable technical variability even within the same lab [54], and informal reports suggest considerable variation between labs and between machines in the same lab. Many of the data analysis issues, which have arisen with microarray technology, may be with us for a long time.

## Practical Steps

If a researcher decides that in fact more expertise is needed in experimental design and subsequent analysis of array data, to whom should she or he turn? Many statisticians are becoming interested in microarray data and may be able to give advice on a project, or mentor a student assistant. Furthermore, there is a great deal of open-source and commercial software for microarrays.

The largest single repository for open-source software is the collection of Bioconductor packages [55], [56] (http://www.bioconductor.org), which are written in the R statistical programming language (http://www.r-project.org). Several courses on data analysis in R using the Bioconductor tools are offered around the world each year. Several commercial software packages (e.g., GeneSpring) now offer interfaces with Bioconductor. There are also several freely available unified suites of software, which include tools for doing many of the functions described here, including, *TM4* (originally produced by The Institute for Genomic Research, and now maintained by John Quackenbush's group at the Dana-Farber Cancer Institute) at http://www.tm4.org; *BRBTools*, maintained by the National Cancer Institute (http://linus.nci.nih.gov/BRB-ArrayTools.html); and *GenePattern* from the Broad Institute (http://www.broadinstitute.org/cancer/software/genepattern/). The Robert S. Boas Center for Genomics and Human Genetics provides a comprehensive survey of free microarray software at http://www.nslij-genetics.org/microarray/soft.html, and Babru Samal maintains a list of free and commercial software at http://www.genetools.us/.

## Supporting Information

Text S1 (0.31 MB DOC)
