# Supplementary material for: Making Informed Choices about Microarray Data Analysis
Source: PLoS Comput Biol. 2010 May 27;6(5):e1000786. doi: 10.1371/journal.pcbi.1000786 (PMC2877726; doi:10.1371/journal.pcbi.1000786)
Supplement: Text S1 — (0.31 MB DOC) [file pcbi.1000786.s001.doc]

**Text S1**


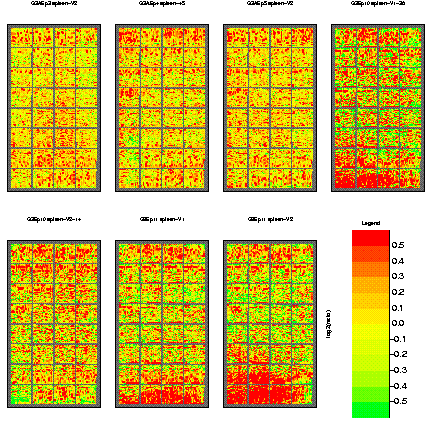

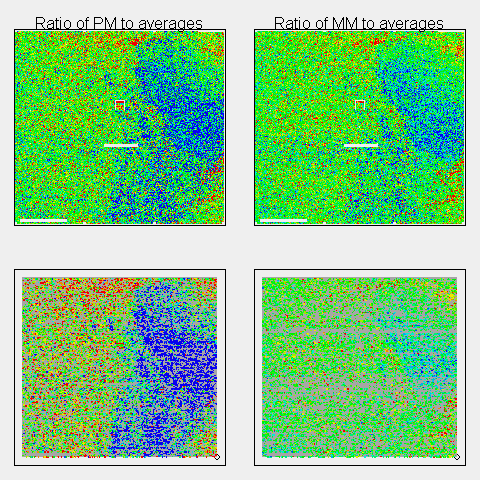

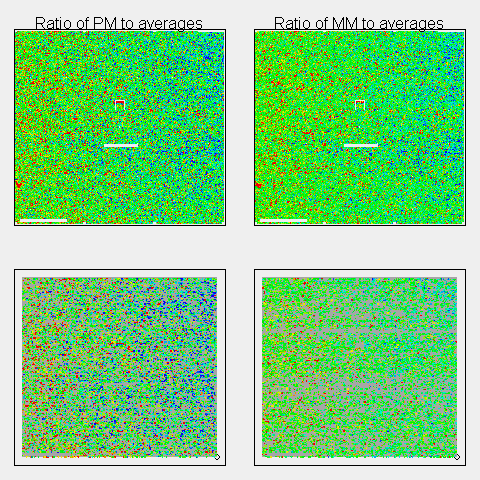

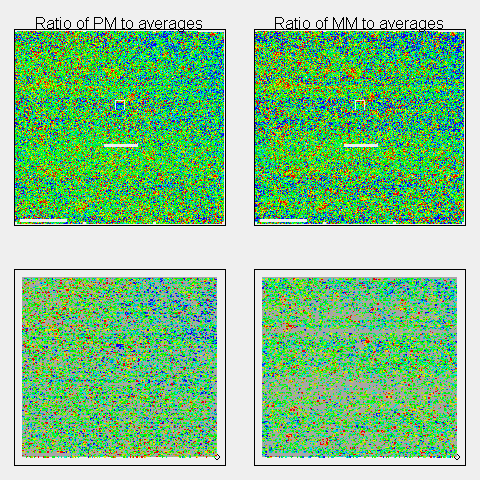


Figure S1.

Top: Spatial images of differences between log R/G ratios on three NCI spotted microarrays and the average of log-ratios for the corresponding probes across nineteen samples from the same tissue. These chips had already passed standard quality control checks at NCI for RNA and hybridization quality (these checks rejected one-third of all chips).

Bottom: Images of log ratios of individual Affymetrix™ Focus chips compared to an average reference chip. Green colors reflect a log-ratio of 0, red a log-ratio of greater than 0.5 and blue a log ratio of less than -0.5. White areas represent non-hybridizing probes. These represent chips GSM25524, 25525 and 25526 from GEO.

**S2 Pre-processing of new (non-expression) array types**

After considerable effort has been expended to find methods to normalize expression arrays, it is disappointing to find that most of these methods are not applicable to most types of non-expression array. The main reason for this is that the crucial assumption of most methods for expression array normalization is that the differences between arrays reflect changes in only a small proportion of the genome and that the overall distribution of expression levels is unchanged. This assumption is certainly not true in other genomic arrays, such as CGH arrays, where samples may differ by orders of magnitude in copy number aberrations, or in ChIP-chips, where overall binding may easily differ by factors of two or three.

**Comparative Genomic Hybridization arrays (CGHa)**

Researchers in cancer and in disease genetics often want to identify regions of the genome that have been duplicated or deleted in a sample. This is often done by comparative genomic hybridisation (CGH) of a sample (in one label) against normal genomic DNA (labelled differently) on an array with probes matching loci distributed fairly uniformly across the genome. The statistical QA methods described for expression arrays will help identify poorly prepared CGH arrays as well. However normalization methods for these arrays are under-developed. The two-channel loess normalization that is commonly applied to two-color expression arrays actually suppresses meaningful changes in two-color CGH arrays. The reason is that the predictor (the supposedly independent variable), which is the average of the two channels, is actually correlated with the supposedly dependent variable (sample copy number), because the reference channel is constant. Although the loess idea for normalization can be more faithfully implemented using the reference channel only, this approach doesn’t deal with the greatest sources of bias in the CGH measures

**Chromatin immuno-precipitation arrays (ChIP-chips)**

Our understanding of epigenetics is expanding at a tremendous rate largely because of the application of ChIP-chips and methylation arrays based on methyl-cytosine immuno-precipitation or restriction enzymes. Although these are two-color arrays, the loess normalization for two-color expression arrays distorts the signals because the average of the two channels is correlated with the signal; furthermore, there are usually two branches to the distribution of log-ratios, which seem to have different characteristics.

The most successful normalizations for ChIP-chips use probe sequence characteristics to try to predict the signal intensities on each chip; the predicted part of the signal is considered to be technical artefact since the probe sequences are unrelated to the biological process being measured. The MAT software package [1] utilizes this idea to normalize Affymetrix™ tiling arrays, but there isn’t a generic package available for most chip types. Furthermore the MAT package builds a statistical model by assuming that only a few probe measures reflect real signal; this assumption is true for arrays assaying transcription factor binding sites, but not for arrays assaying chromatin modifications.

**Single-Nucleotide Polymorphism Arrays (SNP chips)**

Probably the most sophisticated current attempt to model systematic probe-dependent biases in array data is the *CRLMM* methodology for Affymetrix™ SNP arrays, which is instantiated in Bioconductor’s *oligo* package. This methodology estimates probe bias on each chip as function of sequence, SNP pair, and length of the restriction fragment containing the SNP. This approach is very successful; estimates based on the *CRLMM* methodology have better than 99.97% accuracy on the known HapMap samples. For SNP 5.0 and 6.0 arrays, the developers recommend the use of the *crlmm* package, which implements a more efficient version of CRLMM.

1. Johnson WE, Li W, Meyer CA, Gottardo R, Carroll JS, et al. (2006) Model-based analysis of tiling-arrays for ChIP-chip. Proc Natl Acad Sci U S A 103: 12457-12462.
